# Bioprinting Vascularized Constructs for Clinical Relevance: Engineering Hydrogel Systems for Biological Maturity

**DOI:** 10.3390/gels11080636

**Published:** 2025-08-12

**Authors:** Jeonghyun Son, Siyuan Li, Wonwoo Jeong

**Affiliations:** 1 Department of Bioengineering, Stanford University, Stanford, CA 94305, USA; 2 Department of Biomedical Engineering, Ulsan National Institute of Science and Technology (UNIST), Ulsan 44919, Republic of Korea; 3 Medical Center Boulevard, Wake Forest Institute for Regenerative Medicine, School of Medicine, Wake Forest University, Winston-Salem, NC 27104, USA; 4 School of Biomedical Engineering and Sciences, Wake Forest University-Virginia Tech, Winston-Salem, NC 27157, USA

**Keywords:** 3D bioprinting, biomaterials, vascularization

## Abstract

Vascularization remains a critical challenge in tissue engineering, limiting graft survival, integration, and clinical translation. Although bioprinting enables spatial control over vascular architectures, many existing approaches prioritize geometric precision over biological performance. Bioprinted vasculature can be understood as a dynamic and time-dependent system that requires tissue-specific maturation. Within this framework, hydrogel systems act as active microenvironments rather than passive scaffolds. Hydrogel platforms vary from natural matrices and synthetic polymers to bioinspired or stimuli-responsive systems, each offering tunable control over stiffness, degradation, and biochemical signaling needed for vascular maturation. The design requirements of large and small vessels differ in terms of mechanical demands, remodeling capacity, and host integration. A key limitation in current models is the absence of time-resolved evaluation, as critical processes such as lumen formation, pericyte recruitment, and flow-induced remodeling occur progressively and are not captured by static endpoints. Advancements in bioprinting technologies are evaluated based on their capacity to support hydrogel-mediated vascularization across varying length scales and structural complexities. A framework for functional assessment is proposed, and translational challenges related to immunogenicity, scalability, and regulatory requirements are discussed. Such integration of hydrogel-driven biological cues and bioprinting fidelity is critical to advancing vascularized constructs toward clinical translation.

## 1. Introduction

Tissues and organs rely on intricately organized vascular networks to support development, homeostasis, and tissue repair [1]. These networks ensure the delivery of oxygen and nutrients, facilitate waste removal, regulate immune surveillance, and mediate paracrine signaling between stromal and parenchymal cells. Loss or dysfunction of vasculature contributes directly to tissue ischemia, impaired regeneration, and metabolic dysregulation, which are hallmarks of diseases such as myocardial infarction, chronic kidney disease, diabetic ulcers, and cerebral infarction [2]. In regenerative medicine, successful integration of vascular systems is a prerequisite for the survival and long-term function of bioengineered tissues. While large-diameter vessels enable surgical anastomosis and sustained perfusion, microvessels support oxygen exchange, capillary remodeling, and immune cell trafficking [3]. Thus, a physiologically relevant vascular construct must simultaneously recapitulate both macrovascular and microvascular functions in a coordinated and application-specific manner.

To address these challenges, in vitro vascular models have been developed to elucidate endothelial cell behavior, angiogenic signaling pathways, and vascular remodeling dynamics under controlled conditions [4]. These platforms include two-dimensional (2D) endothelial monolayers, three-dimensional (3D) spheroid and organoid systems, microfluidic organ-on-a-chip devices, and hydrogel-based scaffolds embedded with vascular cells [5]. Although these systems offer modularity and reproducibility, many fail to reproduce key in vivo characteristics such as flow-induced shear stress, hierarchical vessel branching, and the complexity of immune and stromal cell interactions. As a result, the translational relevance of these platforms remains limited.

Hydrogel-based systems have emerged as a promising solution, offering biochemical and biomechanical tunability to better emulate native vascular niches [6]. By incorporating extracellular-matrix-derived peptides, angiogenic growth factors, and stromal support cells, hydrogels can promote endothelial adhesion, migration, and stabilization [7,8,9,10]. Controlled degradation kinetics enable timely vascular infiltration and integration, while modulating mechanical properties facilitates the formation of dense capillary networks [11,12]. When systematically engineered, these features allow hydrogels to support the dynamic processes underlying tissue-specific vascularization in both physiological and pathological contexts.

Bioprinting represents a powerful extension of these strategies [13,14,15], enabling the precise spatial deposition of hydrogel-based bioinks containing cells and biochemical cues [16]. This technique allows for the fabrication of vascular networks with application-specific geometry and cellular organization. At the macroscale, bioprinted vessels can be engineered to reduce thrombosis and enable immediate perfusion following implantation [17]. At the microscale, prevascularized tissue constructs support rapid inosculation with host vasculature, improving graft survival and accelerating functional integration [18]. Patient-specific vascular geometries derived from medical imaging data can be faithfully reproduced, enabling the design of anatomically accurate, personalized grafts [10]. Collectively, these capabilities position bioprinting as a central platform for constructing vascularized tissues that are structurally complex, biologically relevant, and clinically scalable.

Despite these advancements, the presence of perfusable vascular structures alone does not ensure functional success. Many printed constructs exhibit regression, leakage, or disorganized vascular architecture, and most evaluations are limited to early static endpoints [19,20]. Functional vascularization must instead be understood as a temporally evolving process that reflects tissue-specific remodeling capacity, integration with host vasculature, and responsiveness to environmental signals. To advance toward clinical application, future strategies must align bioprinting parameters, hydrogel design, and cellular composition with the physiological demands of the target tissue across time. In this review, we highlight the need to reframe vascularization not as a fixed design feature but as a dynamic, functional process. We propose a multidimensional framework for evaluating vascular constructs based on their remodeling potential, long-term integration, and therapeutic relevance, shifting the focus from structural replication to clinically meaningful vascular performance.

## 2. Development and Maturation of Functional Vasculature in Tissue

**Figure 1:**
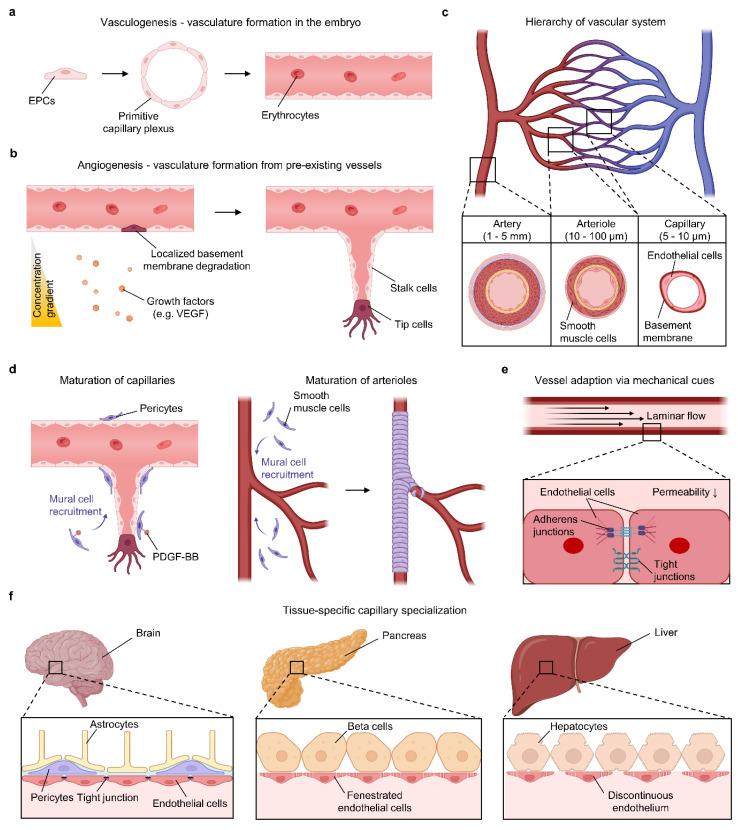
Development and maturation of functional vasculature. (**a**) Vasculogenesis occurs during early embryonic development, wherein endothelial progenitor cells (EPCs) coalesce into a primitive capillary plexus that undergoes lumen formation and stabilization. (**b**) Angiogenesis expands pre-existing vasculature via sprouting, initiated by localized basement membrane degradation and VEGF-driven tip cell migration, followed by stalk cell elongation. (**c**) Structural hierarchy of the vascular system consists of arteries (>100 μm to several mm), arterioles (10–100 μm), and capillaries (5–10 μm), each with distinct wall compositions and functions. (**d**) Vascular maturation involves mural cell recruitment, including pericytes in capillaries and smooth muscle cells in arterioles, which is mediated by PDGF-BB signaling and contributes to vessel stabilization and matrix deposition. (**e**) Mechanical cues, such as laminar shear stress, induce cytoskeletal alignment and junctional reinforcement in endothelial cells, enhancing vessel quiescence and barrier function. (**f**) Tissue-specific specialization results in organ-adapted microvasculature: tight-junction-rich blood–brain barrier in the brain, fenestrated capillaries in the pancreas, and highly permeable sinusoidal vessels in the liver.

The definition of a “functional” blood vessel is inherently context-dependent. A vessel that suffices for nutrient delivery in a dermal graft may be inadequate for cardiac tissue requiring pulsatile flow and tight barrier regulation. Conversely, excessive or abnormal vascularization (as in tumor models) can produce leaky, disorganized vessels that mimic pathological states better than healthy homeostasis. Thus, vascular “function” must be defined relative to the biological and mechanical requirements of each target tissue. Early metrics such as lumen formation and perfusability offer only a static snapshot of a dynamic system. A functional vasculature must not only form but also adapt, mature, and integrate within the evolving tissue environment. Figure 1 summarizes these key stages of vascular maturation, highlighting the cellular and mechanical processes required for vessel formation.

### 2.1. Developmental Mechanisms of Vasculature Formation

In the embryo, blood vessels first arise by vasculogenesis, wherein mesoderm-derived endothelial progenitors to form a primitive capillary plexus [21,22]. Subsequent angiogenesis expands this network, predominantly via sprouting from pre-existing microvessels. Sprouting angiogenesis begins with localized degradation of the vascular basement membrane, pericyte detachment, and loosening of endothelial cell–cell junctions [22,23,24]. Guided by growth factor gradients such as VEGF, a leading tip cell migrates into the surrounding matrix while trailing stalk cells proliferate to elongate the sprout. As two sprouts meet and fuse, a contiguous vessel is formed, and a patent lumen develops. Lumen formation involves coordinated endothelial rearrangements and often intracellular vacuole fusion to hollow out the nascent vessel. Notably, cell–cell adhesion molecules like VE-cadherin are essential for endothelial cells to form stable junctional rings and a central lumen during this process [25,26]. Once an initial capillary network is perfused, blood flow commences, and the vessels begin to remodel.

### 2.2. Structural Hierarchy from Capillaries to Arteries

The mature vascular system exhibits a hierarchical architecture spanning capillaries, arterioles, and arteries, each with distinct structural and functional roles [27,28]. Capillaries are the smallest vessels (5 to 10 μm lumen diameter), consisting of a single endothelial cell layer and a thin basement membrane. This thin-wall structure facilitates efficient molecular exchange with surrounding tissues. Indeed, capillary walls are semipermeable by design and are sufficiently “leaky” to allow nutrient and waste exchange [28,29]. Different tissues employ different capillary subtypes with varying permeability: continuous capillaries have tight endothelial junctions and only small intercellular clefts, whereas fenestrated capillaries have pores in the endothelium (found in absorptive or filtration organs), and sinusoidal capillaries have large openings and discontinuous basement membranes (in bone marrow, liver, spleen) [28,29]. These structural variations reflect the balance between barrier function and exchange capacity needed for each tissue’s physiology.

Arterioles, in contrast, are resistance vessels that control blood flow into capillary beds [27,28]. They range from approximately 5 μm up to 100 μm in diameter and possess a much thicker wall relative to their size, including one to several layers of smooth muscle cells around the endothelium. This muscular layer allows arterioles to constrict or dilate, regulating perfusion pressure and directing blood to specific regions. Arterioles branch into networks of capillaries (often via short transitional metarterioles), and precapillary sphincters (smooth muscle cuffs) further modulate flow into individual capillary loops. Arteries, the largest conduits, have multilayered walls with substantial smooth muscle and elastic fiber content to withstand and even dampen the high, pulsatile pressures generated by the heart. Their thick tunica media and elastic laminae help maintain blood pressure and flow between heartbeats. Unlike capillaries, normal arteries and arterioles are not sites of significant molecular exchange; instead, they serve to transport blood efficiently to the microvasculature. During development, many large vessels emerge from remodeling of the capillary plexus: small microvessels enlarge (arteriogenesis) and recruit additional smooth muscle to become arterioles and arteries. This remodeling is guided by genetic cues such as arterial versus venous differentiation signals and hemodynamic forces, ensuring that a connected hierarchy of large and small vessels forms to meet the tissue’s needs [21].

### 2.3. Vascular Maturation and Stabilization

Following initial vessel formation (by vasculogenesis or angiogenesis), further maturation is required to achieve long-term stability and full functionality [30]. Early-stage capillary sprouts are fragile and highly dependent on external growth factors for survival. A crucial maturation step is the recruitment of mural cells, pericytes in the case of capillaries and small venules, and vascular smooth muscle cells in the case of arterioles and arteries [30,31]. Mural cell recruitment is mediated by signals from endothelial cells such as platelet-derived growth factor-BB (PDGF-BB), which attracts pericyte precursors to nascent microvessels [30]. Indeed, mice lacking PDGF-BB exhibit capillaries largely devoid of pericytes, leading to hemorrhagic and unstable vasculature. As pericytes attach to the endothelial tube, they physically and biochemically support the vessel. This association stimulates endothelial quiescence and initiates deposition of a robust basement membrane matrix. Ultrastructural studies show that a mature basement membrane is often laid down only after endothelial cells and pericytes establish contacts. Similarly, in larger vessels, smooth muscle cells wrap around the endothelium in concentric layers, providing contractile support and secreting extracellular matrix components such as collagen and elastin to fortify the vessel wall.

Concurrently, endothelial cells undergo junctional remodeling to strengthen the vessel’s barrier properties. They upregulate adherens junction proteins (VE-cadherin, β-catenin) and tight junction components (occludin, claudins, ZO-1), which reduce leakiness once the immediate need for growth and sprouting passes. The presence of pericytes profoundly enhances micro-vessel barrier function. For example, pericyte-deficient retinal capillaries become hyperpermeable and prone to abnormal angiogenesis, as seen in diabetic retinopathy. Overall, the physical association between the nascent endothelial tube and mural cells delivers paracrine signals, such as TGF-β activated upon contact, that stabilize the vessel, shifting it from a pro-angiogenic, growth factor-dependent state to a quiescent, durable state. In the absence of this maturation step, new vessels often regress without pericyte or smooth muscle coverage, whereas endothelial tubes remain unstable and can even undergo apoptosis when growth factor support such as VEGF wanes [30,32].

Maturation also involves functional specialization of vessels. Endothelial cells begin to express organ-specific markers and transporters, and vessels acquire the ability to dynamically regulate tone and permeability. For instance, arterioles develop innervation and respond to vasoactive stimuli, while capillaries in the brain induce blood–brain barrier properties (tight junctions, specialized nutrient transport) in concert with astrocytes [33]. This gradual tuning of vascular properties ensures that the network not only survives but operates in harmony with tissue demands.

### 2.4. Role of Mechanical Cues in Vessel Adaptation

Mechanical forces, especially fluid shear stress from blood flow and cyclic stretch from pulse pressure, are pivotal in driving vascular adaptation and maturation. Once a nascent vessel connects to circulation, the blood flow exerts shear forces on endothelial cells [34]. Shear stress stimulates endothelial cells to elongate and align in the direction of flow, reorganize their cytoskeleton, and reinforce cell–cell junctions. Under sustained laminar flow, endothelial junction proteins such as VE-cadherin and occludin are redistributed and stabilized at the cell periphery, tightening the barrier and reducing permeability [34,35]. Experiments show that high shear stress promotes endothelial survival and quiescence, whereas an absence of flow leads to stunted, regressive vessels. Part of this response is mediated by mechano-transduction pathways, such as the PECAM-1/VE-cadherin/VEGFR complex, that sense shear and trigger anti-apoptotic and vessel-stabilizing signals within endothelial cells [35].

In larger vessels, circumferential stretch resulting from pulsatile pressure influences the maturation of smooth muscle layers [36]. Cyclic stretch encourages smooth muscle cells to produce elastin and collagen fibers in an organized manner, contributing to the functional elastic recoil of arteries. Vessels also undergo adaptive remodeling in response to mechanical load [37,38]. For example, if blood flow through a vessel increases chronically, shear-stress-mediated signaling will induce the vessel to enlarge its lumen (outward remodeling) to accommodate higher flow. Conversely, persistently low flow can cause a vessel to narrow or even regress if it is rendered functionally unnecessary. Thus, mechanical cues ensure that the vascular network’s structure is refined post-development, matching vessel caliber, wall thickness, and branching density to the actual hemodynamic demands [37]. Importantly, many of these adaptive processes require time and continued stimuli. Engineered vessels in vitro often remain immature unless exposed to physiological flow conditions, underscoring how critical mechanical factors are for achieving a truly functional vasculature.

### 2.5. Tissue-Specific Vascular Requirements

While all blood vessels share core features, their specific structural and functional attributes vary greatly across different organs and tissue types [39]. Capillary specialization is a prime example. In the systemic circulation, continuous capillaries with small intercellular clefts are ubiquitous, but in the brain, these same capillaries form an exceptionally tight barrier with essentially no clefts, extensive tight junctions, and supporting astrocytic end-feet, collectively constituting the blood–brain barrier [33,39]. In contrast, tissues such as the endocrine glands, intestine, or kidney glomeruli have fenestrated capillaries, which permit rapid fluid and solute exchange to facilitate secretion or filtration [39,40]. The liver and bone marrow push permeability to the extreme, employing sinusoidal capillaries with discontinuous endothelium and large fenestrations that allow proteins and even cells to traverse the vessel wall [39,41]. These differences are not arbitrary; they reflect each organ’s unique balance between permeability and protection. For instance, the brain’s vasculature must strictly limit molecule entry to protect neural tissue, whereas the liver’s sinusoidal vessels must allow plasma proteins and new blood cells to enter circulation freely.

## 3. Scale Matters: Large-Caliber vs. Microvascular Design in the Clinic

The physiological and clinical requirements for vascular constructs differ significantly by vessel diameter (Table 1). Large-caliber vessels, commonly defined as greater than 1 mm in diameter and exceeding 6 mm for clinical graft applications, are essential for conduit-level perfusion and surgical anastomosis [42,43,44]. These include interventions such as coronary artery bypass grafting, arteriovenous (AV) access for hemodialysis, and soft tissue reconstructions including tendon repair or free flap transfer [45,46,47]. In contrast, microvascular networks composed of capillaries, arterioles, and venules (typically less than 100 μm in diameter) mediate nutrient exchange, oxygen diffusion, and immune trafficking at the tissue level [48,49,50]. Each vascular scale imposes distinct functional demands and material constraints that must be addressed in engineered constructs.

### 3.1. Large-Caliber Vessels: Mechanical Integrity and Thromboresistance

Large-caliber vessels must sustain high-pressure pulsatile flow and withstand surgical manipulation. Clinically approved synthetic grafts such as Gore-Tex (expanded polytetrafluoroethylene), Dacron (polyethylene terephthalate), and Carboflo are commonly used in procedures requiring robust vascular conduits [42,51,52]. These materials prioritize non-thrombogenic surfaces, tensile strength, and suture retention [53]. However, they often lack cellular integration, which can result in intimal hyperplasia, occlusion, or chronic inflammation [54]. To prevent these complications, engineered large vessels must achieve burst pressures exceeding 2000 mmHg, maintain long-term patency (over 85 percent at 12 months), and resist immune-mediated rejection [55]. Endothelial cell seeding improves hemocompatibility but fails to fully replicate the anti-inflammatory and remodeling functions of native endothelium [56,57]. Co-culture with smooth muscle cells or perivascular fibroblasts may provide mechanical and paracrine support, yet challenges remain in controlling layer-specific organization and avoiding fibrotic overgrowth [58,59].

In hemodialysis patients, AV grafts must often be replaced due to complications such as thrombosis, stenosis, and infection. Nearly 20 to 30% of AV grafts fail annually, requiring replacement surgery [60]. One study estimated that AV grafts undergo interventions at a mean rate of 0.24 events per year, with substantial variability depending on patient comorbidities. Moreover, the annual cost for AV graft maintenance in the United States ranges from $60,000 to $70,000 per patient, which exceeds the cost associated with AV fistulas [61]. These data underscore the need for durable, immunologically compatible constructs that can reduce the burden of repeated surgeries and long-term catheter dependence. The unmet clinical need persists for bioengineered alternatives that combine hemocompatibility, mechanical durability, and endothelialization capacity. Emerging strategies include bioinert luminal coatings, heparin bonding, and modular scaffolds that allow staged cell integration post-implantation. Furthermore, tendon reconstruction and composite flap transplantation, which require vascularized soft tissue with suturable caliber, highlight the demand for patient-specific large vessel constructs capable of supporting immediate perfusion upon implantation.

### 3.2. Microvascular Networks: Prioritizing Remodeling and Integration

Microvascular structures govern the viability of engineered tissues by facilitating metabolic exchange and paracrine signaling. Clinical models such as Integra Dermal Regeneration Template and the Sernova Cell Pouch demonstrate the necessity of prevascularization in skin and islet transplantation, respectively [62,63]. These constructs require highly porous, degradable matrices that promote angiogenic sprouting and inosculation with host vasculature. Functional integration typically demands inosculation within 3 to 5 days to prevent hypoxia-related necrosis, especially in constructs exceeding 200 to 300 μm in thickness. Unlike large vessels, microvasculature prioritizes remodeling capacity over mechanical strength and therefore relies on proangiogenic cues, dynamic extracellular matrix interactions, and cellular responsiveness. Capillary-sized constructs (typically 5 to 100 μm in diameter) must achieve lumen stability under low-pressure flow, permit immune cell trafficking, and adapt to local metabolic demands. Hydrogel systems with tunable stiffness, VEGF gradients, and stromal co-culture techniques, such as inclusion of pericytes or adipose-derived stromal cells, are frequently employed to promote stable and responsive microvascular formation [64,65,66].

However, excessive or disorganized angiogenesis can be detrimental [67,68]. In some constructs, uncontrolled sprouting leads to tortuous or leaky vessels that compromise perfusion efficiency and structural integrity. These pathological microvessels, resembling those in tumor environments, may exacerbate inflammation, hinder graft integration, or induce fibrotic encapsulation. Therefore, strategies that guide vessel alignment, stabilize junctional complexes, and regulate angiogenic signaling are critical for functional success. Importantly, the immunological profile of microvessels must balance rapid host integration with minimal inflammatory activation, which remains a key bottleneck in the clinical success of vascularized grafts for applications such as diabetic wound healing or intraparenchymal cell therapy.

### 3.3. Hierarchical Integration Strategies

In clinical settings, engineered tissues often require both large-caliber conduits for surgical anastomosis and fine microvascular networks to sustain tissue viability. For example, in composite tissue reconstruction such as flap grafts, a surgically connectable artery or vein must be paired with perfusable capillaries to ensure oxygen delivery and waste removal throughout the graft volume [69,70,71,72]. This dual requirement presents a translational challenge: how to develop vascular architectures that span distinct length scales while maintaining functional integration. Neither vascular scale alone is sufficient. Large vessels without downstream capillaries can result in perfusion mismatch, central necrosis, and tissue failure. Conversely, microvessels without upstream flow input cannot maintain physiological perfusion or respond to dynamic circulatory demands [73,74,75]. Successful hierarchical integration requires constructs that couple macro-scale conduits with dense microvascular beds in a way that preserves pressure gradients, flow continuity, and metabolic responsiveness.

Functionally, such constructs must demonstrate high-pressure tolerance at the proximal end, gradual pressure attenuation through branching architecture, and active remodeling capacity in distal regions. Spatial continuity of the endothelium, controlled luminal diameter transitions, and biomechanical compatibility across scales are essential to ensure stable perfusion and prevent flow disruption, thrombosis, or edema [39,76,77]. Furthermore, hierarchical constructs must support synchronous maturation of vessel subtypes and enable host integration at both surgical and microvascular interfaces. These requirements highlight the need for design principles that prioritize functional interdependence between vessel scales, rather than focusing on anatomical replication alone.

## 4. Hydrogels as Dynamic Vascular Niches

### 4.1. Matrix Composition and Biochemical Cues

**Figure 2:**
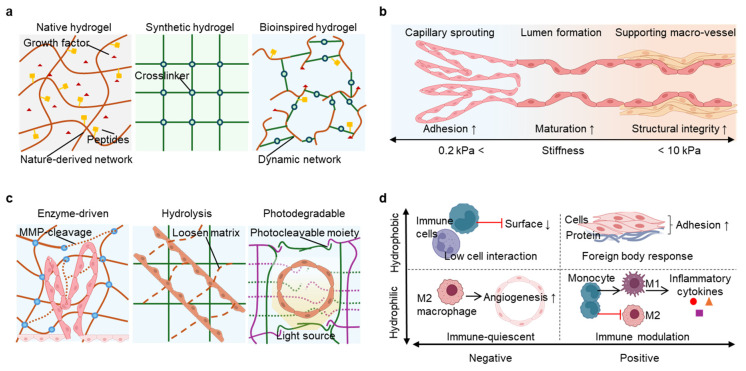
Key design principles of hydrogels for vascularized tissue engineering. (**a**) Comparison of synthetic and native-inspired hydrogels. Native-like matrices contain intrinsic biological cues, including growth factors and peptides, that enhance endothelial interaction and angiogenesis. Synthetic materials provide tunable mechanical properties with minimal bioactivity. (**b**) Hydrogel stiffness, controlled through crosslinking and polymer architecture, regulates endothelial migration, sprouting, and tubulogenesis. Soft hydrogel supports cell adhesion while stiff hydrogel increases structural integrity, indicated with arrows. Matching stiffness to vessel scale supports both macrovascular stability and microvascular branching. (**c**) Matrix degradation via enzymatic cleavage, hydrolysis, or photodegradation modulates porosity, stiffness relaxation, and vascular invasion. (**d**) Surface chemistry, including hydrophilicity and surface charge, influences immune cell behavior and vascular remodeling. Hydrophilic and negatively charged surfaces promote anti-inflammatory macrophage responses and angiogenesis. As indicated with up or down arrows, hydrophobic or positively charged surfaces often induce inflammation, fibrosis, and impaired vascular integration by down regulating cell interaction, up regulating cell adhesion, or promoting M1 macrophage release cytokines.

Native hydrogels, such as collagen, fibrin, and the dECM (decellularized extracellular) matrix, offer the most biomimetic environments for vasculature formation [78]. These materials inherently contain integrin-binding domains like the arginine–glycine–aspartic acid (RGD) sequence, proteoglycans, and matrix-bound growth factors that promote endothelial cell adhesion, migration, and sprouting (Figure 2a, Table 2). Their microstructure and high porosity in polymeric network allow cell infiltration, oxygen diffusion, and matrix remodeling, initiating angiogenesis under physiological conditions. For example, fibrin promotes early tube formation through integrin-mediated cytoskeletal reorganization, while dECM preserves organ-specific ECM and growth factors such as angiogenic cues [78,79]. In a recent study, researchers’ incorporation of fibrin into cardiac ECM hydrogels enhances their angiogenic properties, inducing robust endothelial cell tube formation and promoting mesenchymal stem cell spheroid sprouting [80]. However, these natural materials often suffer from poor mechanical tunability and batch variability, limiting reproducibility and scalability.

To address these limitations, synthetic hydrogels offer better control and versatility in tunability, biocompatibility, and injectability such as PEG (polyethylene glycol), PVA (polyvinyl alcohol), and Pluronic derivatives that offer precise control over mechanical properties and chemical functionality [78,85,95]. Although bioinert by default, synthetic hydrogels can be functionalized with short adhesive peptides, such as RGD, to support endothelial attachment and tubulogenesis. Additionally, growth-factor-binding motifs can be incorporated to localize pro-angiogenic signals. Heparin is a highly sulfated and negatively charged polysaccharide that, when conjugated to synthetic hydrogel networks, enables electrostatic sequestration of positively charged growth factors like VEGF (vascular endothelial growth factor), FGF-2 (fibroblast growth factor 2), and insulin-like growth factor [96]. This interaction enhances local retention, stabilizes protein conformation, and enables gradient formation for directional vascular sprouting. PEG-heparin composites exemplify this strategy, enabling both spatial control and sustained delivery of bioactive factors. Furthermore, the hydrogel system can be designed to possess shear-thinning property and temperature responsiveness, which offers adjustable delivery and in situ gelation, expanding their clinical usefulness [97].

Bio-inspired hydrogels are designed to emulate key features of the native vascular microenvironment while offering tunable physical properties. These systems integrate adhesive ligands such as RGD with synthetic backbones that allow precise control over crosslinking and stiffness. For instance, GelMA (gelatin methacrylate) retains cell-adhesive sequences from gelatin and enables light-based patterning for spatial control [98]. One of hydrogel systems is specifically designed to respond to cell-driven remodeling processes, which will be discussed in detail in the following sub sections. Rather than simply combining materials, bio-inspired hydrogels aim to replicate adaptive tissue-like behavior, including angiogenic factor presentation and mechanosensitive feedback.

### 4.2. Stiffness and Mechanotransduction

Designing hydrogels with appropriate mechanical properties is essential for guiding vascular morphogenesis, as endothelial cells sense and respond to stiffness, viscoelasticity, and matrix resistance through integrin-mediated mechanotransduction (Figure 2b) [99]. These cues are sensed through integrin engagement, focal adhesion formation, and cytoskeletal contractility, which collectively activate downstream signaling pathways that govern endothelial migration, alignment, tubulogenesis, and junctional stability. Importantly, the stiffness requirements of hydrogels differ markedly depending on the vascular scale. Hydrogel stiffness significantly influences cell proliferation and differentiation Different matrix stiffness support different cell fates. Soft matrices (<0.2 kPa) support the endothelial differentiation and microvascular formation, and stiff (>10 kPa) matrices impair barrier function, increase vessel permeability, and can be used to print the formation of large lumen [100,101,102].

To evaluate the impact of mechanical properties on cell behavior and tissue regeneration, many researchers utilize different hydrogel formulations in their studies. A recent study showed there was 28.5-fold more branching and 4.9-fold greater network volume fraction in a 0.19 kPa soft matrix, compared with 0.88 kPa matrix [103]. In addition, there was a stiffness window around 5 to 10 kPa for promoting capillary-like networks, maximizing both filopodia activity and tubule stability. These responses are mediated by mechanotransduction pathways including YAP/TAZ signaling (Yes-associated protein/transcriptional coactivator with PDZ-binding motif), Notch, and RhoA/Rho kinase (ROCK), which transduce cytoskeletal tension into gene expression changes that guide endothelial behavior [91,104,105,106]. Recent computational and experimental models have highlighted that stiffness values near 9.8 kPa produce the most robust vascular networks, indicating a sharp threshold for angiogenic activation [91]. Engineered hydrogels enable precise tuning of stiffness through adjustments in polymer concentration, crosslinking density, and network architecture [91]. Stiff matrices (from 10 to 60kPa) are usually used in vascular formation in hard tissues or pathological environments, for example, 17.5kPa to 44.6kPa supports revascularization in bone defects than lower or higher stiffness matrix, and tumor angiogenesis through integrin-β3-dependent mechanotransduction pathways [107].

Synthetic systems such as PEG-diacrylate, GelMA, and HAMA (hyaluronic acid methacrylate) allow fine modulation of the elastic modulus and stress relaxation properties. Moreover, spatial patterning of stiffness using techniques like photopatterning or gradient crosslinking enables the replication of physiological tissue interfaces, including transitions from perivascular zones to parenchymal regions [108,109]. Dynamically stiffening hydrogels provide additional control by allowing vascular invasion under initially soft conditions, followed by enzyme-mediated stiffening to support long-term vessel maturation [84,85]. This spatiotemporal control is particularly relevant in bioprinting applications, where mechanical zones must align with the vascular architecture being reconstructed. By aligning hydrogel stiffness with the functional and structural requirements of different vessel types, engineered constructs can better recapitulate the mechanical environment of native tissues and support scalable, perfusable, and clinically relevant vascularization.

### 4.3. Degradability and Remodeling Compatibility

Degradability is a key design parameter for vascularized hydrogels, enabling the matrix to evolve in response to cellular activity and support dynamic processes such as vessel sprouting, expansion, and tissue integration (Figure 2c) [11,85,110]. Unlike static scaffolds, degradable systems allow endothelial and stromal cells to dynamically reshape their environment, facilitating matrix invasion, tubulogenesis, and long-term stabilization of vascular networks. The mode and rate of degradation must be tailored to match the kinetics of vascular remodeling. Excessively rapid degradation may result in structural collapse or leakage, while overly slow breakdown can hinder angiogenic progression and delay host integration [111]. A widely used approach involves incorporating matrix metalloproteinase (MMP)-cleavable peptide sequences into the hydrogel backbone [85]. These motifs enable enzyme-mediated degradation in response to MMPs secreted by endothelial cells and associated stromal populations. In PEG-based systems, MMP-sensitive crosslinkers provide spatially localized degradation that aligns with cellular activity, allowing for vessel infiltration and branching without compromising the mechanical stability of the entire construct. By designing MMP-sensitive crosslinkers with either one or two cleavage sites, the hydrogel can have different degradability and further benefit cell secreted ECM deposition [85]. The incorporation of microgels and MMP-sensitive linkers creates a balance of porosity and degradability control, thereby regulating endothelial cell invasion gated network stability, and host integration [112]. Through strategic biochemical functionalization with RGD and VEGF-mimetic peptides, these materials bolster endothelial cell adhesion, migration, and sprouting, as well as provide rapid and enduring vascularization for regenerative therapies [113]. This cell-responsive degradability has been shown to enhance the formation of interconnected capillary networks and support tissue-specific remodeling in vitro and in vivo.

In addition to enzyme-driven remodeling, hydrogel degradation can proceed through hydrolysis of labile bonds or photo-responsive cleavage. Widely used materials like hyaluronic acid and gelatin naturally degrade over time through hydrolysis, with rates that can be adjusted by modifying the crosslink density or chemical structure. These systems provide predictable, time-dependent softening of the matrix, which can enhance neovessel expansion and perivascular integration [11]. Hydrolytic degradation also enables mass transport of nutrients and signaling molecules throughout the scaffold, particularly in dense or cell-rich constructs. Photodegradable hydrogels add another layer of control, allowing user-directed matrix remodeling through light exposure. Polymers functionalized with photocleavable moieties (e.g., HA-tyramine or PEG-based linkers) can be locally degraded in response to specific wavelengths [87,88]. This capability enables precise modulation of porosity, stiffness, or network topology at defined time points or regions. In bioprinted systems, photodegradation has been used to create perfusable channels, release embedded angiogenic factors, or dynamically adjust tissue architecture during maturation [86,89,90].

Another approach is using pH-responsive hydrogel for printing delicate vascular structures. A recent study used the gallic-acid-functionalized and methacrylated hyaluronic acid hydrogel’s pH-responsive nature that allows for precise control over viscosity at slightly basic conditions (pH 7.5–8), facilitating smooth extrusion and high-fidelity filament creation. This customizable gelation is especially well-suited for bioprinting vascular networks straight onto tissue surfaces since it also facilitates shape preservation during printing and encourages in situ stabilization in physiological settings [114]. Accordingly, these engineered features collectively offer greater control and reproducibility compared to the inherent limitations of native ECM materials.

To meet the dual challenge of promoting early vascular invasion while maintaining long-term structural support, many advanced hydrogel designs employ dual network strategies. The double network system can combine two hydrogels with different degrading rates, mechanical integrities, and network densities to meet the dual challenge. A fast-degrading, soft, and low-density hydrogel network can promote rapid cell entry and proliferation, and on the other hand, the dual network system also has a stable, stiff, and high-density hydrogel network that can maintain shape and mechanical integrity over printing, extended culture, or implantation. In a related study, GelMA and HAMA were used together with hyaluronidase to fine-tune the mechanical properties of the printed construct post-fabrication [115]. Such staged degradation frameworks are particularly relevant for volumetric tissue constructs where synchronized remodeling and scaffold persistence are both required [6,115]. By coupling degradation kinetics to the biological pace of angiogenesis and tissue remodeling, engineered hydrogels can transition from inert supports to responsive environments that guide vascular integration. When appropriately tuned, degradable matrices support both the initiation and the long-term function of engineered vasculature, advancing their utility in regenerative medicine and translational applications.

## 5. Bioprinting Strategies for Scalable Vascularized Tissue Construction

**Figure 3:**
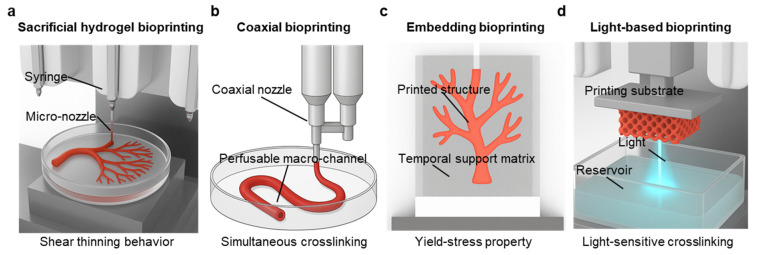
Bioprinting strategies for fabricating vascularized constructs. (**a**) Multi-nozzle extrusion printing enables direct deposition of hierarchical vascular structures within Petri dishes using bioinks through micro-nozzles. (**b**) Coaxial printing facilitates the fabrication of perfusable hollow macro-channels using a dual-syringe coaxial nozzle system. (**c**) Embedded printing allows branched vascular structures to be fabricated within a temporal support matrix, enabling high-resolution and spatially complex constructs. (**d**) Light-based bioprinting enables layer-by-layer fabrication of intricate micro-structure using a photo-crosslinkable hydrogel system.

Engineering vascular structures often begins with fabricating simple perfusable channel-like structures. Among various vascularization methods, bioprinting uniquely offers precise spatial control, enabling hierarchical, integrated channel-like structures as physiological vascular networks [116]. Achieving clinically meaningful outcomes with bioprinting depends not only on resolution or bioink (typically hydrogel-based formulations) biocompatibility but also on scalability and physiologically relevant geometries supporting long-term perfusion [117]. Interconnected capillaries are essential for efficient mass transport, yet direct printing of true capillary networks remains challenging [118]. To address this, bottom-up strategies such as the self-assembly of endothelial cells or angiogenic remodeling have recently been combined with bioprinting techniques to engineer integrated capillary networks in situ [90]. These biologically driven capillaries are known to remodel through lumenization and enhance mass transport capacity [119]. Integrating such bottom-up mechanisms into bioprinted constructs therefore offers a promising route to achieve fine-scale vascularization within engineered tissues [120]. Thus, bioprinting approaches include sacrificial printing, coaxial extrusion, embedded printing, and light-based bioprinting, each with distinct advantages and limitations, along with varying degrees of compatibility for integration with bottom-up approaches (Figure 3, Table 3).

### 5.1. Sacrificial Hydrogel Extrusion-Based Bioprinting

**Figure 4:**
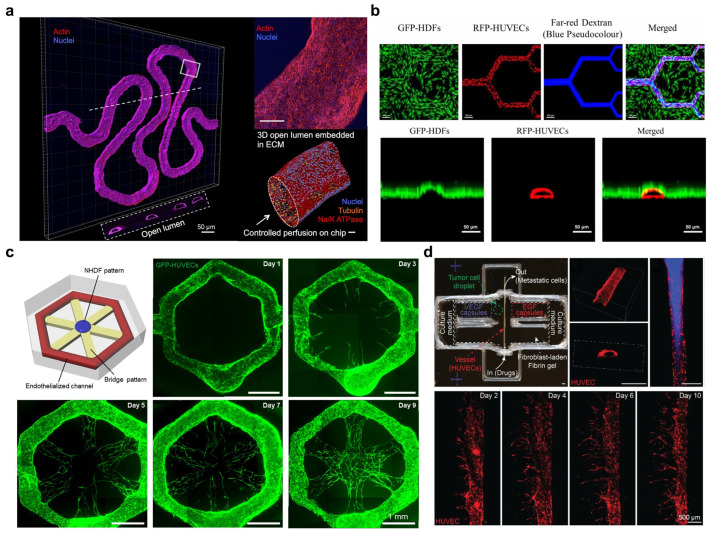
Engineering continuous vascular networks via sacrificial hydrogel extrusion-based bioprinting. (**a**) A 3D-printed convoluted proximal tubule model with open lumens that is embedded in the ECM. Image adapted from Homan et al. [124], under CC BY 4.0. (**b**) High-resolution electrohydrodynamic inkjet printing of microvascular patterns (RFP-HUVECs) surrounded by fibroblasts (GFP-HDFs). Image adapted from Zheng et al. [132], under CC BY 4.0. (**c**) Hepatic-lobule-like microvasculature with self-organized capillary networks formed via a bottom-up approach. Image adapted with permission from Son et al. [18], © 2021 Wiley-VCH. (**d**) A 3D bioprinted tumor-on-a-chip model with angiogenic capillaries forming over time via VEGF capsules within fibrin gel. Image adapted with permission from Panoskaltsis-Mortari et al. [163], © 2019 Wiley-VCH.

Extrusion-based sacrificial bioprinting is a widely used technique for fabricating perfusable channels due to its simplicity and compatibility with multi-nozzle systems. In this method, a fugitive ink such as gelatin, Pluronic F127, or a sugar-based gel is printed into a supporting matrix and encapsulated by a crosslinkable hydrogel [13,20,121,122,123]. Subsequent removal of the sacrificial ink (e.g., via melting or dissolution) yields hollow channels suitable for perfusion and endothelial lining [121,124,125]. This approach enables scalable fabrication under mild processing conditions and produces continuous channel structures with geometric complexity that mimic native vasculature (Figure 4a) [20,121,122,123,124,125]. Endothelialization of the channels is a key step, with two main strategies reported. The first involves removing the sacrificial material, then introducing endothelial cells into the resulting lumens via perfusion or injection [121,122,123,124,125]. This method allows precise control over cell type and timing but may result in uneven coverage in narrow or tortuous channels, particularly in vertically oriented or deeply embedded regions. Flow conditioning, starting with low shear stress through bioreactors, is often necessary to promote uniform endothelial cell adhesion and maturation [126,127,128].

An alternative approach involves pre-loading endothelial cells into the sacrificial ink [18,129,130]. Upon removal, these cells deposit along the channel surface, enabling more uniform seeding in complex geometries [129,130]. This method facilitates early matrix–cell contact but imposes stricter requirements on the fugitive ink, including cytocompatibility and suitable rheological properties. It also limits spatial control over cell distribution, as all embedded cells follow the print path. Thus, this strategy improves uniformity at the expense of flexibility. Despite its scalability, sacrificial bioprinting remains limited by resolution [90,116,119,120]. Printable channel diameter is typically constrained to 100–500 µm due to nozzle size, ink viscosity, and extrusion stability. Channels near the lower end often suffer from collapse or clogging. To address this, high-resolution techniques such as electrohydrodynamic (EHD) printing and melt electrowriting have been applied. EHD-assisted printing enables sacrificial filaments as small as ~30 µm (Figure 4b) [131,132], while melt electrowriting (MEW) with thermoresponsive polymers has achieved microchannels below 100 µm with high fidelity [162]. After printing and cooling, the polymer fibers could be dissolved to leave behind branching channels that follow physiological design principles (e.g., Murray’s law of branching). These refinements show that extrusion-based sacrificial hydrogel approaches can be pushed toward smaller feature sizes, but true capillary-scale lumens (<20 µm) remain out of reach for direct writing in most cases. Moreover, these methods still yield only 2.5D patterns on the substrate rather than truly three-dimensional vascular networks.

Another ongoing challenge is the incorporation of functional microvessels into hierarchical vascular networks. While extrusion-based sacrificial printing can generate channels with diameters around 100 µm, capillaries in the 10–20 µm range are typically formed through cellular self-organization rather than direct printing [119,120]. To address this, printed microchannels can be used as larger parent vessels that promote capillary formation in the surrounding matrix (Figure 4c,d) [18,163,164]. When endothelial cells, supportive stromal cells such as fibroblasts or pericytes, and pro-angiogenic signals are embedded in the matrix, the presence of a perfused channel can guide nearby cells to sprout new capillary structures and establish connections with the printed vessels. However, this approach relies on complex and variable cell behaviors, which can lead to incomplete vascular integration, unstable lumen formation, or regression of nascent vessels [120]. Continued progress of extrusion-based printing techniques in improving resolution, optimizing fugitive ink properties, and supporting long-term perfusion and remodeling is needed to enable robust, multiscale vascularization in engineered tissues.

### 5.2. Coaxial Bioprinting: One-Step Fabrication of Multi-Layered Vessels

**Figure 5:**
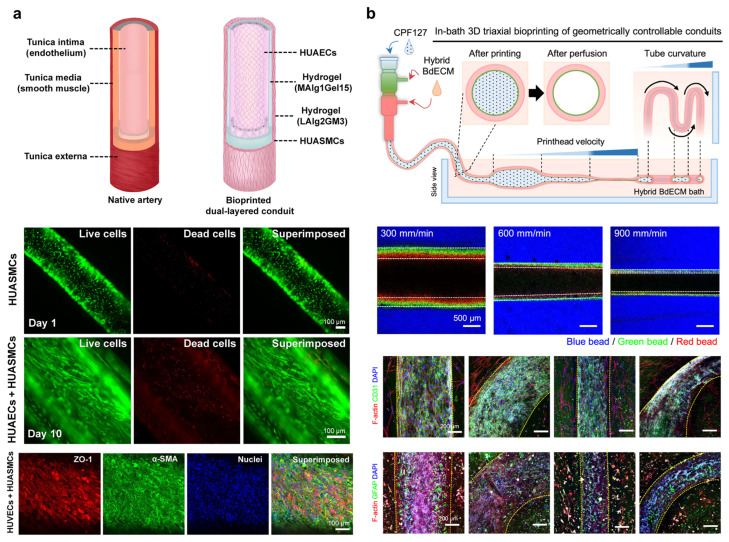
Multi-layered vessel fabrication via coaxial bioprinting. (**a**) Fabrication of bioprinted arterial conduits composed of an outer layer of smooth muscle cells (HUASMCs) and an inner endothelial lining (HUAECs). Image adapted with permission from Wang et al. [6], © 2022 American Association for the Advancement of Science (AAAS). (**b**) The 3D bioprinted multilayered cerebrovascular conduits with tunable geometry and astrocyte-embedded surroundings. Image adapted from Park et al. [165], under CC BY 4.0.

Coaxial bioprinting is a versatile extrusion-based technique for fabricating tubular structures by extruding multiple bioinks concentrically through a specialized multi-channel nozzle [133,134,135]. This method was originally developed to replicate the multi-layer architecture of native blood vessels (Figure 5a) [6,136,137,138,139,140], where an inner endothelial layer, a middle smooth muscle layer, and an outer adventitia work together to provide proper vascular function. Ionic or thermal gelation can occur immediately when the core and sheath bioinks interact with their respective crosslinking agents during co-extrusion at the tip of the coaxial nozzle. The result is a continuous hollow filament with a tunable lumen diameter and wall thickness, produced in a single extrusion step [136,139]. This concentric printing allows a one-step creation of perfusable tubes with multiple layers. Compared to sacrificial printing, coaxial extrusion enables immediate lumen formation without any post-printing removal step. This eliminates the need for dissolving a fugitive template and thereby reduces the risk of structural distortion [141]. It also means the printed vessel can be perfused with flow as soon as printing is complete [139]. The crosslinked sheath serves as a built-in supporting wall, which helps the tube hold its shape and can guide cells to align circumferentially similar to how smooth muscle cells orient in real vessels. The throughput and relative simplicity of this approach have made it popular for applications ranging from engineered vascular grafts to in vitro vascular models [135,137,139,142,143,144,145,146,165].

Coaxial bioprinting has shown feasibility in fabricating large-diameter vascular conduits suitable for surgical implantation [6,138,139]. Small-caliber grafts containing multiple cell layers have been printed by embedding smooth muscle cells or fibroblasts in the outer wall and endothelial cells along the lumen [6,139]. These constructs exhibited mechanical properties, such as burst pressure resistance and suture retention, sufficient to withstand physiological blood flow, and were successfully anastomosed into the circulation of small animal models [138,139]. In rodent studies, the printed vessels maintained patency after implantation into sites such as the abdominal aorta or vena cava. These findings demonstrate the potential of coaxial bioprinting to generate functional vascular grafts capable of physiological integration.

To simulate microphysiology in vasculature, coaxial printing was applied to organ-on-a-chip systems and in vitro disease platforms [142,143,144,145,146,165]. In these settings, the focus shifts from mechanical strength to mimicking vascular function at smaller scales. Sub-millimeter-diameter microvessels produced via coaxial printing have been integrated into microfluidic devices to study endothelial barrier integrity, inflammation-driven remodeling, and cancer extravasation. Recent work showed that stenosis-mimicking vascular constructs can be fabricated using embedded 3D-coaxial bioprinting with mechanically reinforced bioinks utilizing a support bath, demonstrating a powerful platform for in vitro disease modeling of flow-mediated vascular pathologies [146]. This strategy was further extended to reconstruct a cerebral vascular microenvironment incorporating astrocytes and to model cancer extravasation mechanisms influenced by vascular geometry (Figure 5b) [165].

Despite its advantages, coaxial bioprinting faces several critical limitations that hinder its broader applicability. The method remains highly dependent on alginate as the primary sheath material [147,148]. Although alginate enables rapid gelation, its lack of cell-adhesive motifs and inherent stiffness limit endothelial cell spreading, migration, and monolayer formation. Strategies to improve cytocompatibility, such as blending alginate with GelMA or collagen, or coating the lumen with extracellular matrix proteins like fibronectin or laminin, have shown benefits but often compromise printability and structural integrity [147,148]. Replacing alginate with cell-interactive biomaterials can improve cellular responses but introduces slower gelation and reduced lumen stability. In addition to material challenges, coaxial printing lacks sufficient resolution to produce capillary-scale lumens below 50 µm [148,166]. Flow instability and nozzle design constraints make direct printing of such fine channels inconsistent. A key challenge is replicating physiologic zonation within printed vessels [148]. While dual-layer structures are feasible, constructing multilayered architectures with distinct and functional boundaries remains difficult within the lumen size. Coaxial printing also limits microvascular integration, as the outer sheath can hinder both capillary ingrowth via bottom-up assembly and inosculation with host tissue in vivo. Addressing these issues in structure, resolution, and integration is essential for clinical translation.

### 5.3. Embedded Bioprinting: Enabling Complex Vascular Architectures with 3D Hierarchy

**Figure 6:**
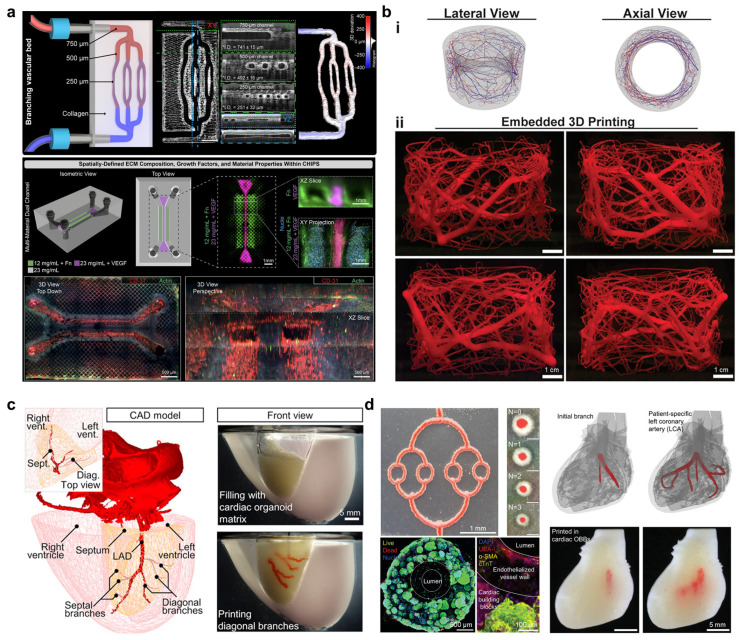
The 3D complex vascular architectures via embedded bioprinting. (**a**) Embedded bioprinting of high-resolution, perfusable collagen-based scaffolds for functional tissue assembly. Image adapted from Shiwarski et al. [151], under CC BY 4.0. (**b**) Fabrication of organ-scale vascular networks using embedded bioprinting guided by artificial-intelligence-assisted design. Image adapted with permission from Sexton et al. [19], © 2025 AAAS. (**c**) Generation of high-density cardiac tissues with physiologically branched vasculature using Sacrificial Writing into Functional Tissue (SWIFT) printing. Image adapted with permission from Skylar-Scott et al. [15], © 2019 AAAS. (**d**) Coaxial-embedded sacrificial writing to fabricate perfusable, artery-scale branches within cardiac tissues. Image adapted with permission from Stankey et al. [159], © 2024 Wiley-VCH.

Embedded bioprinting offers a distinct strategy in which bioinks are extruded into a temporary support matrix, often referred to as a suspension bath, rather than into air [149]. This matrix, typically composed of jammed microgel slurries or granular hydrogels such as gelatin microparticles, physically stabilizes printed filaments until they solidify. The approach enables freeform fabrication of complex 3D structures, including overhangs and branching channels that would otherwise collapse. A representative example is the FRESH (Freeform Reversible Embedding of Suspended Hydrogels) technique, which uses a gelatin microparticle bath to support bioinks like collagen, fibrin, or Matrigel, materials that are otherwise too low in viscosity for standard extrusion [150]. The yield-stress properties of the bath allow precise deposition of fine filaments, which can be crosslinked in situ and later retrieved by melting the support matrix. Embedded bioprinting has achieved vascular constructs with channel diameters in the 50 to 100 µm range, approaching microvessel-scale resolution (Figure 6a) [151,152,153,154]. Using this method, researchers have fabricated coronary-artery-like networks with tortuous geometries and multilevel branches that remain perfusable by blood analogs [155]. To further enhance vascular complexity at the organ scale, a recent approach used artificial-intelligence-driven design and embedded printing to fabricate sophisticated branched vascular networks (Figure 6b) [19]. Because the support bath constrains filaments in all directions, the vascular hierarchies can be printed in a single, continuous construct. Additionally, this technique accommodates fragile ECM-based hydrogels, such as type I collagen and decellularized matrix derivatives, without requiring synthetic thickeners [156,157,158]. By eliminating the need for self-supporting viscosity, embedded printing enables construction of biologically relevant architectures that promote cell viability and tissue-specific function.

Despite these strengths, embedded bioprinting comes with its own set of challenges and trade-offs [150,160,161]. One practical consideration is the need for tight control of the support bath’s rheological properties. The bath must behave like a solid to support the ink during printing but then yield and flow away when the construct is recovered. Inconsistencies in bath composition, temperature, or printing speed can lead to defects. For instance, if the bath is too fluid, structures may drift or sag; if too stiff, the nozzle may have trouble extruding ink or may compress the already printed filaments. Maintaining uniform conditions throughout a large volume is non-trivial, especially for long print times or thicker tissues. Another major limitation is scalability. Constructing organ-sized tissues requires sequential deposition of fine structures throughout the entire volume, which is time-consuming due to the layer-by-layer process. In addition, post-print removal of the support matrix must be performed with care to preserve structural integrity, further increasing processing time. These factors constrain both the maximum construct size and overall high throughput fabrication.

In addition to FRESH, which enables freeform printing of soft hydrogels within a support bath, other embedded bioprinting strategies have been developed to address scalability. A prominent example is SWIFT (Sacrificial Writing into Functional Tissue), where sacrificial inks are printed directly into a preformed tissue matrix, such as a dense slab of organoids or spheroids embedded in a soft gel [15]. The printed channels are later evacuated, leaving perfusable conduits within the living tissue. This approach allows rapid vascularization of thick, cell-dense constructs by integrating vascular networks post hoc, and it has been applied to centimeter-scale cardiac tissues to prevent central necrosis (Figure 6c) [15]. Unlike traditional embedded printing, SWIFT operates without an external bath and can preserve native-like cellular architecture while enabling perfusion [15]. Recent adaptations have incorporated coaxial sacrificial inks, forming multilayered vessels within bulk tissues and suggesting a path toward hierarchical vascular integration (Figure 6d) [159]. However, SWIFT remains limited in resolution. Sacrificial channels are typically hundreds of micrometers wide, as finer nozzles can clog or disrupt surrounding cells. As a result, SWIFT is suited for mid-sized vessels, while capillary-scale networks still rely on post-printing cellular self-assembly, which remains a major challenge in thick constructs [149,159]. This trade-off between scalability and fine resolution highlights the need for hybrid strategies that bridge macro- and microvascular systems.

Despite these limitations, embedded bioprinting approaches such as FRESH and SWIFT have enabled the fabrication of anatomically complex, perfusable tissues, including cardiac, pulmonary, and hepatic models [102,155]. Future developments in support material rheology, printhead precision, and multi-material deposition are expected to improve resolution, increase throughput, and expand the range of printable tissue architectures. Recent work has introduced embedded printing strategies not only to improve resolution but also to guide endothelial self-organization into capillary-scale networks within printed vessels, reflecting continued efforts in embedded printing research to achieve integrated microvasculature [151]. Embedded bioprinting is poised to play a pivotal role in bridging the gap between engineered macro-vessels and emergent capillary networks, bringing fully vascularized, clinically relevant tissues within reach.

### 5.4. Light-Based Bioprinting: High-Resolution Patterning of Capillary-Scale Vasculature

**Figure 7:**
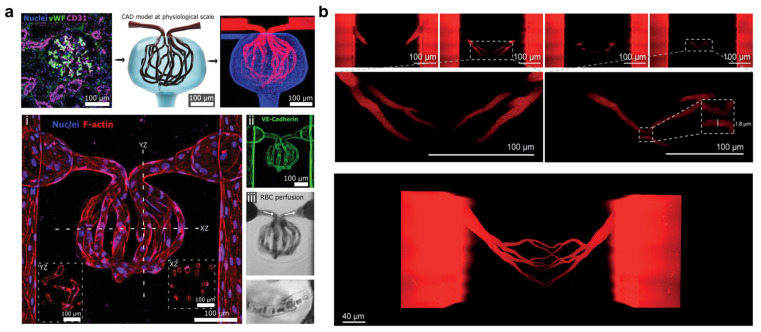
High-resolution capillary-scale vascular patterning via light-based bioprinting. (**a**) Projection-stereolithography-based bioprinting of glomerulus-mimetic vascular structures with endothelialized, perfusable capillary networks at physiological scale. Image adapted with permission from Rayner et al. [169], © 2021 Wiley-VCH. (**b**) Two-photon polymerization of perfusable, capillary-like bifurcating vascular channels with subcellular resolution. Image adapted from Rizzo et al. [172], under CC BY-NC 4.0.

Light-based bioprinting techniques, including digital light processing (DLP), stereolithography (SLA), and two-photon polymerization (2PP), use patterned light to selectively crosslink photocurable hydrogels with high spatial precision [167]. These methods offer the highest resolution among current bioprinting strategies, enabling direct fabrication of vascular structures at capillary scales of 10 to 20 µm, without relying on bottom-up strategies (Figure 7) [86,168,169,170,171,172]. DLP and SLA polymerize entire layers in parallel, while 2PP enables voxel-level patterning within a 3D volume, allowing the construction of complex geometries such as branched or tortuous capillary networks. These techniques produce smooth-walled, anatomically accurate channels with minimal flow disturbance. For example, DLP has been used to fabricate vascular lattices and tree-like structures with diameters of 50 to 100 µm, while 2PP has produced networks as small as 10 µm [173]. In addition, these methods minimize shear stress during printing, as cells are encapsulated in photocurable bioinks and patterned under gentle light exposure, supporting high cell viability.

Despite these advantages, light-based bioprinting faces important material-related constraints. Photocurable bioinks must be chemically modified with reactive groups such as methacrylates, which can alter their biological performance [174]. Commonly used polymers like PEG-diacrylate and highly concentrated GelMA often lack cell-adhesive ligands or are too stiff to mimic native ECM [175]. Photoinitiators, which initiate crosslinking upon light exposure, can be cytotoxic at high concentrations or under prolonged illumination [176]. Balancing efficient crosslinking with cytocompatibility remains a challenge. GelMA is widely used because it provides moderate bioactivity and good printability, but it still requires UV or blue light and photoinitiators such as LAP or Irgacure that must be carefully optimized to preserve cell function [175,176,177].

Scalability remains a challenge, particularly for large constructs. While DLP and SLA offer efficient layer-by-layer fabrication, limited light penetration and overcuring can reduce resolution in deeper regions. These effects can be mitigated by incorporating UV blockers and adjusting the refractive index of the resin to confine polymerization and reduce optical distortion [178]. Although 2PP enables high-resolution 3D patterning, its slow writing speed limits scalability [179]. Emerging volumetric methods such as computed axial lithography (CAL) may improve throughput, though they are still in early development [149,178,180]. Another major challenge is the endothelialization of photo-patterned microscale channels. Perfusion-based seeding is largely ineffective in channels below 20 µm due to high flow resistance and clogging [86]. Alternative strategies include co-printing sacrificial-cell-laden filaments that dissolve post-printing, photopatterning channels within a pre-seeded matrix, or functionalizing channel walls with bioactive ligands such as RGD peptides or collagen fragments to promote in situ cell migration. Without a functional endothelial lining, microchannels lack critical features such as selective permeability and anti-thrombogenicity.

## 6. Future Perspectives: Hydrogel System and Bioprinting Techniques to Engineer Physiologically Relevant Vasculature

Bioprinting technologies continue to evolve as a powerful top-down approach for fabricating vascular networks with hierarchical architecture and clinical scalability. While early studies were constrained by the poor printability of soft, biocompatible hydrogels, more recent strategies have improved the extrusion of softer, native-like hydrogels by incorporating viscosity-enhancing hydrogels composed of gelatin, hyaluronic acid, or xanthan gum [181]. Embedded bioprinting has further expanded the material palette by supporting low-modulus inks within yield-stress baths, enabling the fabrication of complex and delicate structures [149]. In parallel, advances in ECM-based hydrogels have led to techniques that allow direct printing of unmodified biological matrices [182,183,184,185]. A particularly notable method employed macromolecular crowding to induce rapid self-assembly of type I collagen, enabling isolation of organized collagen μ-bundles and their incorporation into high-resolution vascular structures [185]. These developments signal a shift from achieving basic printability to engineering biomimetic vascular systems with structural and biochemical fidelity.

Application of bottom-up strategies has shown considerable promise in forming integrated capillary networks within bioprinted constructs [90]. However, sustaining the long-term stability of these capillary structures remains a major challenge [120]. One key factor is the precise spatiotemporal control of angiogenic signals, as vascular sprouting and maturation are governed by distinct molecular cues [186]. Fine-tuned delivery of pro-angiogenic factors will be critical for promoting vascular integrity and remodeling. Another important aspect is the microfluidic environment: while interstitial flow generally supports angiogenesis [126,187,188], excessive luminal shear stress has been shown to inhibit capillary formation [189], suggesting a physiological threshold beyond which flow becomes detrimental [190]. Notably, most insights into these mechanisms have been derived from 2.5-dimensional microfluidic models, and how such principles apply to large-scale, fully 3D-printed constructs remains poorly understood. Future studies should explore how dynamic biochemical and mechanical microenvironments can be spatially and temporally orchestrated to promote stable vascular integration.

Another hurdle of bottom-up strategies is the time needed to establish lumenized and perfusable capillaries, often five days or more, which may not be fast enough to support cell viability in thick tissues immediately after printing [191]. This delay may be insufficient to support cell viability in thick constructs immediately after printing. To overcome this gap, emerging hybrid strategies aim to introduce predefined capillary-scale voids into cell-dense hydrogels [163,192,193]. Four-dimensional bioprinting using stimuli-responsive hydrogel systems that dynamically transform in response to external cues such as pH, temperature, or light offers emerging opportunities for creating hollow vascular structures with real-time adaptability [194]. These engineered channels guide endothelial migration and inosculation, offering an alternative to relying solely on angiogenic or vasculogenic-like self-organization. These strategies represent a potentially promising solution for accelerating microvascular integration and facilitating the transition from structural design to functional maturity.

## 7. Conclusions

Hydrogels serve as the foundational matrix for vascular bioprinting. Their tunable biochemical and mechanical properties enable spatial organization, mechanical support, and biological signaling critical for vascular development. These characteristics allow hydrogels to replicate key aspects of the extracellular matrix and support cell viability, differentiation, and tissue integration. Based on these advantages, the application of hydrogel systems in bioprinting must be guided by functional, time-resolved metrics rather than static structural outcomes. Key endpoints include patency under physiological flow, rapid inosculation (≤5 days) with host vasculature, barrier integrity, and immune acceptance, especially in perfused tissues such as skin, pancreas, or muscle. Long-term studies should evaluate not only vascular morphology but also the influence of printed vessels on adjacent parenchymal differentiation and metabolic function. To meet these criteria, integration with closed-loop bioreactor systems is further essential. These platforms enable continuous perfusion, mechanical stimulation, and monitoring of vascular integrity and remodeling kinetics. In particular, vascular constructs must retain stability under cyclic load while promoting endothelial quiescence and mural cell recruitment, mimicking native vessel maturation.

However, achieving reproducible, scalable production remains a bottleneck. Bioprinted vascular grafts require large quantities of GMP-compliant vascular cells, including endothelial cells and pericytes, which may necessitate industrialized iPSC-derived cell manufacturing. Moreover, high-throughput, automated printing platforms must be developed for parallelized construct fabrication with integrated quality control checkpoints. Compatibility with high-throughput screening systems could enable rapid evaluation of graft responses to drugs or inflammatory signals under patient-matched conditions. The immunological profile of vascular constructs also plays a critical role in their clinical viability. Material composition, degradation products, and architecture can modulate macrophage polarization, fibrotic responses, and vascular regression. Advanced immune-modulatory hydrogels, incorporating ligands or cytokines, offer routes to promote tolerance while supporting angiogenesis.

Finally, translation into clinical workflows requires collaboration with surgeons and hospital systems. Constructs must be implantable within operative time constraints, allow vascular anastomosis, and integrate into existing monitoring frameworks. Regulatory alignment through preclinical validation in large-animal models, followed by GMP-standardized production, will be essential for human use. Ultimately, bioprinted vasculature must satisfy not only biological design requirements but also clinical, logistical, and regulatory demands. Addressing these multi-layered challenges will be pivotal for moving beyond proof-of-concept studies toward real-world regenerative therapies.

## Figures and Tables

**Table 1:** Distinct engineering requirements of macro- and microvascular systems.

Feature	Large-Caliber Vessels	Microvascular Networks
Diameter	>1 mm (≥6 mm for grafts)	<100 μm
Primary Role	Bulk perfusion, anastomosis	Oxygen/nutrient exchange
Key Functional Metrics	Patency, burst pressure	Lumen stability, remodeling
Clinical Risks	Clotting, intimal hyperplasia	Leakiness, perfusion failure
Material Needs	High strength, hemocompatible	Degradable, cell-responsive
Example Applications	Coronary bypass, AV grafts, tendon/flap repair	Skin grafts, islet implants

**Table 2:** Hydrogel design parameters for supporting vascular function.

Functional Role	Attribution	Hydrogels
Endothelialization	Adhesion, spreading, lumen formation	Fibrin, collagen, GelMA, dECM [80,81]
Bioactivity	RGD, heparin, angiogenic factor binding	RGD-GelMA, heparin-GelMA, fibrin [78,79]
Mechanical Properties	Stiffness, viscoelasticity, and mechanical resistance	PEGDA blends, GelMA composites, methacrylated ECM [82,83,84,85]
Degradation Compatibility	MMP-cleavable, hydrolytic, photodegradable	GelMA, HA-tyramine, PEG-MMP [86,87,88]
ECM Mimicry	Alignment, porosity, matrix composition	Collagen, fibrin, dECM [86,89,90,91].
Surface Chemistry Immune Modulation	Zwitterions, charge, macrophage polarization, reduce foreign body reaction	PVA, zwitterionic hydrogels, sericin [92,93,94]

**Table 3:** Comparison of 3D bioprinting technologies for vascular engineering.

Strategy	Resolution	Scalability	Material Versatility	ECM Fidelity	Compatibility with Bottom-Up Strategies	References
Sacrificial Printing	Moderate (~100–500 µm)	Moderate–High	High–wide ink compatibility	Moderate–High	High	[13,20,121,122,123,124,125,126,127,128,129,130,131,132]
Coaxial Extrusion	Low (~500–1000 µm)	High: continuous strand, rapid	Limited: mainly alginate-based materials	Low–moderate: limited ECM mimicry	Low	[6,133,134,135,136,137,138,139,140,141,142,143,144,145,146,147,148]
Embedded Printing	High (≤20 µm)	Moderate–High: Scalable but time-intensive	Very High: compatible with soft ECM bioinks	High: native-like composition and stiffness	Moderate–High	[15,19,149,150,151,152,153,154,155,156,157,158,159,160,161]
Light-Based Printing	Very High (≤10 µm)	Low–Moderate: high speed but small volume	Low: constrained to photopolymerizable inks	Low–Moderate: depends on photoink tuning	Not necessary	
