# Correction: Son et al. Bioprinting Vascularized Constructs for Clinical Relevance: Engineering Hydrogel Systems for Biological Maturity. *Gels* 2025, *11*, 636

**DOI:** 10.3390/gels11090719

**Published:** 2025-09-09

**Authors:** Jeonghyun Son, Siyuan Li, Wonwoo Jeong

**Affiliations:** 1 Department of Bioengineering, Stanford University, Stanford, CA 94305, USA; 2 Department of Biomedical Engineering, Ulsan National Institute of Science and Technology (UNIST), Ulsan 44919, Republic of Korea; 3 Medical Center Boulevard, Wake Forest Institute for Regenerative Medicine, School of Medicine, Wake Forest University, Winston-Salem, NC 27104, USA; 4 School of Biomedical Engineering and Sciences, Wake Forest University-Virginia Tech, Winston-Salem, NC 27157, USA

The authors would like to make the following corrections to [1]. The changes are as follows:

**Missing citations**

In the original publication, the last row of Table 3 had citations missing. We have added the references’ citations in the last row of the corrected Table 3.

**Table 3:** Comparison of 3D bioprinting technologies for vascular engineering.

Strategy	Resolution	Scalability	Material Versatility	ECM Fidelity	Compatibility with Bottom-Up Strategies	References
Sacrificial Printing	Moderate (~100–500 µm)	Moderate–High	High–wide ink compatibility	Moderate–High	High	[13,20,121–132]
Coaxial Extrusion	Low (~500–1000 µm)	High: continuous strand, rapid	Limited: mainly alginate-based materials	Low–moderate: limited ECM mimicry	Low	[6,133–148]
Embedded Printing	High (≤20 µm)	Moderate–High: Scalable but time-intensive	Very High: compatible with soft ECM bioinks	High: native-like composition and stiffness	Moderate–High	[15,19,149–161]
Light-Based Printing	Very High (≤10 µm)	Low–Moderate: high speed but small volume	Low: constrained to photopolymerizable inks	Low–Moderate: depends on photoink tuning	Not necessary	[86,162–175]

The updated references’ citations Refs. [162–175] in the original are Refs. [167–180], Refs. [176–180] in the original are Refs. [162–166], we have changed the order of references and have adjusted accordingly. The authors state that the scientific conclusions are unaffected. This correction was approved by the Academic Editor. The original publication has also been updated.

## References

[1] Son J., Li S., Jeong W. (2025). Bioprinting Vascularized Constructs for Clinical Relevance: Engineering Hydrogel Systems for Biological Maturity. Gels.

